# Research on rural domestic sewage discharge, influencing factors and pollution loads in the Yangtze river basin

**DOI:** 10.1038/s41598-025-24747-5

**Published:** 2025-11-20

**Authors:** Junchao Li, Lifang Wang, Zheng Liu, Ting Cao, Haiyang Lü, Yao Zhu, Xuhua Li

**Affiliations:** 1 State Key Laboratory of Environmental Criteria and Risk Assessment, Chinese Research Academy of Environmental Sciences, Beijing, 100012 China; 2 Environmental Development Center of the Ministry of Ecology and Environment, Sino-Japan Friendship Centre for Environmental Protection, Beijing, 100029 China; 3 SEPA Key Laboratory of Eco-Industry Chinese Research Academy of Environmental Sciences, Beijing, 100012 China

**Keywords:** Yangtze river basin, Rural domestic sewage, Equivalent pollution load, Correlation analysis, Environmental sciences, Hydrology

## Abstract

In this study, the characteristics of rural domestic wastewater discharge in the Yangtze River Basin, China, are analysed via geographic information visualization, pollutant load assessment, and correlation analysis. Through the use of a geographic information visualization system, this study intuitively presents the characteristics of rural domestic wastewater discharge in the Yangtze River Basin. In pollutant load assessment, the pressure caused by rural domestic wastewater discharge on maintaining the target water quality standards in the tributaries and main stream of the Yangtze River is comprehensively analysed. Correlation analysis reveals the social and natural factors influencing the levels of rural domestic wastewater discharge. The findings indicate that the average discharge level of rural domestic wastewater in the Yangtze River Basin remains low, with an average water discharge level of 39.24 L/(capita·day), a chemical oxygen demand (COD) of 27.50 mg/(capita·day), an ammonia nitrogen (NH_3_-N) content of 1.53 mg/(capita·day), a total nitrogen (TN) amount of 2.74 mg/(capita·day), and a total phosphorus (TP) content of 0.22 mg/(capita·day). Based on the current levels of rural domestic wastewater discharge and the concentrations of the above substances in the natural environment, the impact of rural domestic wastewater discharge on maintaining water quality functions in the tributaries and main stream of the Yangtze River can be considered negligible, although regional differences exist. Discharge levels are significantly influenced by various social factors, including educational level, per capita disposable income, and consumption expenditures, as well as natural factors such as average annual temperature, average annual humidity, and average annual rainfall. Overall, this study provides reference data for the analysis and management of rural domestic wastewater discharge in both similar regions in China and globally.

## Introduction

 Owing to the constraints imposed by social development trends, the rural ecological environment and the investment in rural environmental infrastructure have consistently lacked effective attention from various sectors of society, and there is insufficient in-depth research on rural sewage treatment efforts, which has left rural environmental governance facing a lack of suitable governance models and regulatory strategies^1–4^. Rural domestic sewage is among the significant sources of nonpoint source pollution in developing countries, and the indiscriminate discharge of rural domestic sewage, which contains large amounts of nutrients such as nitrogen (N) and phosphorus (P), as well as pollutants such as detergents and pathogenic microorganisms, has exacerbated rural water pollution issues, particularly water quality eutrophication and the presence of black and odorous water bodies, potentially posing a threat to the safety of drinking water. This situation not only jeopardizes the ecological safety and landscape sustainability in rural areas but also adversely affects rural food production, food safety, and public health^5–9^. The issue of rural domestic sewage is directly related to the quality of the rural ecological environment, regional drinking water safety, maternal and child health, and food supply security^6,10,11^. China is not only a populous country but also an agricultural giant; according to data, by the end of 2023, the permanent rural population in China reached 477 million people, accounting for approximately 33.81% of the total national population, with the rural domestic sewage treatment rate reaching only 45%. However, the pollutants entering water bodies are derived primarily from rural nonpoint source pollution, with rural domestic sewage being a significant component of this type of pollution^12,13^.

In recent years, with the advancement of rural living environment improvement initiatives in China, various universities and research institutions have continuously increased their focus on engineering technologies and theoretical frameworks for rural domestic wastewater treatment. Research on rural domestic sewage has gradually increased^14–16^. Existing studies have revealed that the limitations of the widely adopted combination of biological and ecological technologies are becoming increasingly notable. The overall governance framework for rural wastewater treatment urgently requires further research and supplementation, key technological breakthroughs are needed, and a greater understanding of the spatial distribution characteristics of rural wastewater and the factors influencing its discharge is needed, as well as long-term observations of potential ecological risks^7,17–20^. The governance of rural domestic sewage still requires theoretical research and support from key parameters, which can effectively increase the accuracy of pollution prevention and control in rural areas and provide a scientific reference for the formulation of rural domestic wastewater management measures.

The Yangtze River Basin represents the largest river basin in China, encompassing 19 out of the 32 provinces (municipalities and autonomous regions) of the country. According to the Statistical Yearbook, the resident population within this basin is approximately 998 million people, accounting for approximately 70% of the total national population. The average urbanization rate in the basin exceeds 60%, but there are significant differences in urbanization rates across different subbasins. With the continuous development of the social economy, the distribution of the rural resident population and rural living habits have undergone significant changes, and rural ecological environmental issues have become increasingly prominent. Research has indicated that the provinces and cities within the Yangtze River Basin face increasing pressure to reduce domestic sewage discharge^21,22^. Human activities in rural areas continually influence the balance of ecosystem services, and the conflict between rural domestic wastewater issues and rural ecosystem services has become one of the key factors constraining coordinated regional development. This directly influences one-third of China’s freshwater resources, three-fifths of the hydropower resource reserves, and the drinking water safety for approximately 400 million people in the middle and lower reaches of the Yangtze River, as well as China’s water usage, agricultural production, tourism development, industrial development, and the sustainable development of living environments.

## Materials and methods

### Study subjects

This study focuses on the largest river basin in China, i.e., the Yangtze River Basin, and covers parts of 15 provinces (municipalities and autonomous regions), including Qinghai Province, the Tibet Autonomous Region, Sichuan Province, Yunnan Province, Gansu Province, Shaanxi Province, Henan Province, Guizhou Province, Chongqing Municipality, Anhui Province, Jiangsu Province, Zhejiang Province, Fujian Province, the Guangxi Autonomous Region, and Shanghai Municipality, as well as all the areas of Jiangxi Province, Hubei Province, and Hunan Province. In this study, the levels and characteristics of rural domestic sewage discharge within this region, the pressure exerted by rural domestic sewage on maintaining water quality objectives in the tributaries and the main stream of the Yangtze River, and the effects of social and natural factors on rural domestic sewage discharge levels in this area are investigated.

### Data sources and processing

The levels of rural domestic sewage discharge in each city (prefecture, state, and league) within the Yangtze River Basin are derived from the Handbook of Statistical Methods and Coefficients for Emission Sources, which was produced by the Ministry of Ecology and Environment of China. This handbook clarifies the methods for calculating rural domestic pollution sources: Rural domestic sewage discharge = permanent rural population × per capita sewage discharge level × 365. Moreover, the discharge of rural domestic sewage pollutants is calculated on the basis of the permanent rural population and the per capita pollution generation intensity, where pollutant generation = permanent rural population × per capita sewage discharge coefficient × 365. In addition, the handbook provides the rural domestic sewage discharge levels in each city (prefecture, state, and league). The relevant website is https://www.mee.gov.cn/xxgk2018/xxgk/xxgk01/202106/t20210618_839512.html.

Data on the permanent rural population and educational status originate from the China Statistical Yearbook (2022) and the 2022 statistical yearbooks of the provinces (autonomous regions and municipalities) in the Yangtze River Basin, as well as from the National Economic and Social Development Statistical Bulletin and the County-Level Data from the China Population Census. The relevant website is https://www.stats.gov.cn/sj/ndsj/.

Data on the per capita disposable income and per capita consumption expenditures are sourced from the China Rural Statistical Yearbook (2021, 2022), the China Regional Statistical Yearbook available at https://www.quyushuju.com/portal.php?mod=list&catid=116, and the 2022 statistical yearbooks and National Economic and Social Development Statistical Bulletins of the provinces (autonomous regions, municipalities) in the Yangtze River Basin. The relevant website is https://www.stats.gov.cn/sj/ndsj/.

Average annual temperature and average annual precipitation data are obtained from the National Oceanic and Atmospheric Administration (NOAA) National Centers for Environmental Information (NCEI). The average annual temperature is based on the latitudes and longitudes of the meteorological stations and daily average temperature values, which are obtained through interpolation (using inverse distance weighting) and are based on national-level city administrative boundary data and temperature raster-based statistics. The average annual precipitation values are compiled from daily precipitation data obtained from all meteorological observation stations in China from 1981 to 2023; data are obtained from stations within China, and average annual precipitation data are derived from meteorological stations nationwide. The relevant website is https://www.ncei.noaa.gov/data/global-summary-of-the-day/archive/.

Average annual humidity data are generated from the processed daily value dataset V3.0 of China’s ground climate data, thereby using Python for data cleaning, point expansion, reprojection, and inverse distance weighting interpolation.

The target water quality data for the tributaries of the Yangtze River are sourced from the Handbook on the Functional Zoning of Important Rivers and Lakes in the Country. Except the Taihu Lake system, the functional target water quality for the secondary rivers and main stream of the Yangtze River is classified as Class II, while the target water quality for the Taihu Lake system is provisionally classified as Class III on the basis of the average actual water quality conditions over the past two years.

### Analytical methods

Kriging interpolation is an advanced interpolation method based on the theory of spatial autocorrelation, fundamentally centred on analysing the spatial distribution patterns of known sampling points to generate a continuous attribute surface for unknown regions, which provides error estimates. This method not only facilitates the predictions of attribute values at unknown points but also quantifies the uncertainties associated with these predictions. The global Moran’s index is used to determine the spatial autocorrelation of sewage discharge across China^23^, with the levels of rural domestic sewage discharge in the Yangtze River Basin derived from discontinuous data corresponding to 123 administrative regions (cities, prefectures, and leagues) within the basin. Preliminary assessments using SPSS confirm that the sample size meets the prerequisite conditions for the kriging interpolation method, i.e., the sample size is greater than 80, and the data exhibit a normal distribution. The geostatistical wizard tool in ArcGIS (version 10.8; ESRI, RL, USA) is employed to apply the kriging interpolation method, enabling a visual representation of the characteristics and disparities in the spatial distribution of rural domestic sewage discharge in the Yangtze River Basin of China.

Spearman’s correlation analysis is a nonparametric method that does not require the original variable distribution to meet specific assumptions. Particularly when resolving nonnormally distributed or ordinal data, Spearman correlation analysis remains effective for assessing the monotonic relationships among variables. In this study, the statistical analysis module of SPSS (version 27.0; IBM, NY, USA) is employed to explore the overall dispersion and central tendency of rural domestic sewage discharge in the Yangtze River Basin, thus providing a detailed understanding of the sewage discharge levels. Spearman correlation analysis was conducted using SPSS to identify closely related factors between the volume and quality of sewage discharge by rural residents and various social and natural factors^24–26^.

Pollution load of rural wastewater: “Load Pollution Equivalent” is a core evaluation indicator used to quantify and compare the degree of environmental impact of pollutants. By standardizing the “actual pollutant emissions” against the “environmental quality standards (or emission standards)” for that pollutant, it eliminates the differences in “toxicity, hazard thresholds, and concentration units” among various pollutants, thereby achieving a “unified scale comparison” for multiple pollutants. This provides a scientific basis for identifying the contributions of regions or pollution sources. Referring to the assessment and evaluation methods of agricultural nonpoint source pollution loads and combining the average annual rainfall in the watershed, the total annual pollution load of pollutants in the water body is calculated using the discharge coefficient method. Finally, the standardized pollution load method is employed for a comprehensive evaluation of various pollutants^27–30^.

1 $$Ki=\frac{{Pi}}{{\sum {Pi} }} \times 100\%$$

2 $$\text{Specific equations} : Pi=\frac{{Qi}}{{Coi}} \times 3.65 \times {10^6}$$

where $Pi$ is the standardized pollution load of pollutant *i*; $Qi$ is the annual average concentration of pollutant *i*, mg/L; $Coi$ is the water quality control standard value for pollutant *i* based on water environment functional zoning, mg/L; and $Ki$ is the ratio of the standardized pollution load of pollutant *i*.

## Results and discussion

### Analysis of emission characteristics

#### Comprehensive characteristics of wastewater emission in the Yangtze river basin

**Fig. 1:**
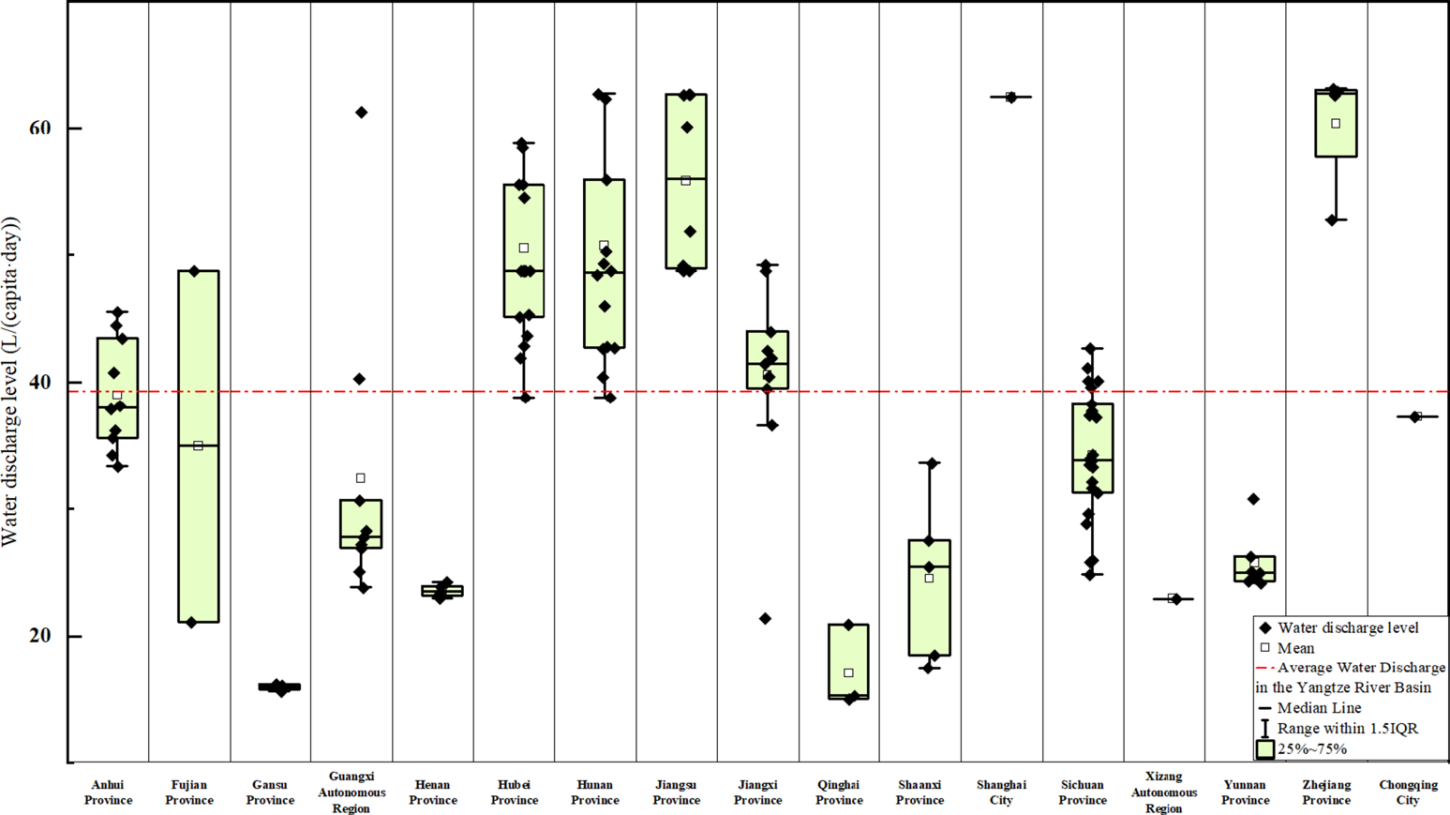
Domestic wastewater discharge volumes from various provinces in the Yangtze River Basin.

**Fig. 2:**
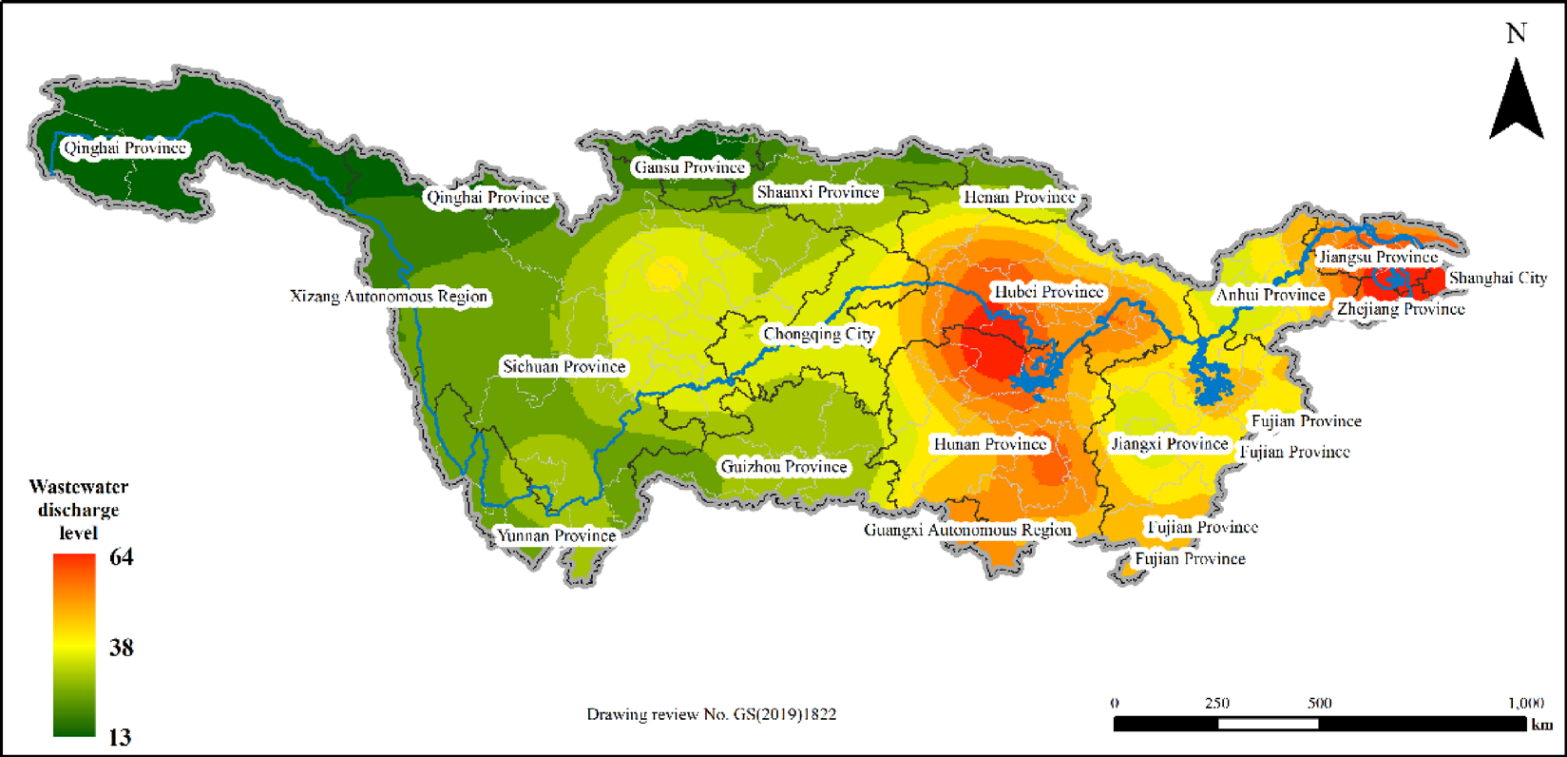
Spatial characteristics of rural domestic sewage discharge in the Yangtze River Basin.

As shown in Fig. 1, the average level of rural domestic sewage discharge in the Yangtze River Basin of China is slightly greater than the national average, with the discharge range essentially conforming with the national level. In most regions, the levels of rural domestic sewage discharge are relatively low. Statistical analysis results are shown in Fig. 2, which reveals that the lowest rural domestic sewage discharge level in the Yangtze River Basin reaches only 15.01 L/(capita·day), which is also the lowest sewage discharge level in the rural areas of China, while the highest is 78.79 L/(capita·day), far below the urban sewage discharge levels in China, which range from 140 to 400 L/(capita·day). The highest rural domestic sewage discharge level is close to the national average of rural domestic sewage discharge of 79.30 L/(capita·day), indicating that the rural domestic sewage discharge level in the Yangtze River Basin can, to some extent, reflect the rural domestic sewage discharge level in China. The average level of rural domestic sewage discharge in the Yangtze River Basin of China is 39.24 L/(capita·day), which is slightly greater than the national average of 37.52 L/(capita·day). In comparison to the discharge levels of 100 to 150 L/(capita·day) observed in Europe and other high-income countries, as well as the urban sewage discharge levels within China, this figure is considered relatively low^31–34^. Furthermore, the value is lower than the sewage discharge levels reported in other developing countries, such as Indonesia, which average 45 L/(capita·day)^35^. Percentile values and dispersion analysis reveal that the rural domestic sewage discharge levels in various cities in the Yangtze River Basin are distributed mainly between the first and third quartiles, with a mean standard error of 1.24 L/(capita·day), a variance of 190.33 L/(capita·day), and a standard deviation of 13.80 L/(capita·day). These findings indicate that the differences in the rural domestic sewage discharge levels among cities in the Yangtze River Basin are relatively small, with low volatility and high concentration and a relatively small overall range, and that the statistical analysis of the mean discharge levels has high reliability and precision, further illustrating the low rural domestic sewage discharge levels in the Yangtze River Basin.

#### Comprehensive water quality emission characteristics in the Yangtze river basin

**Fig. 3:**
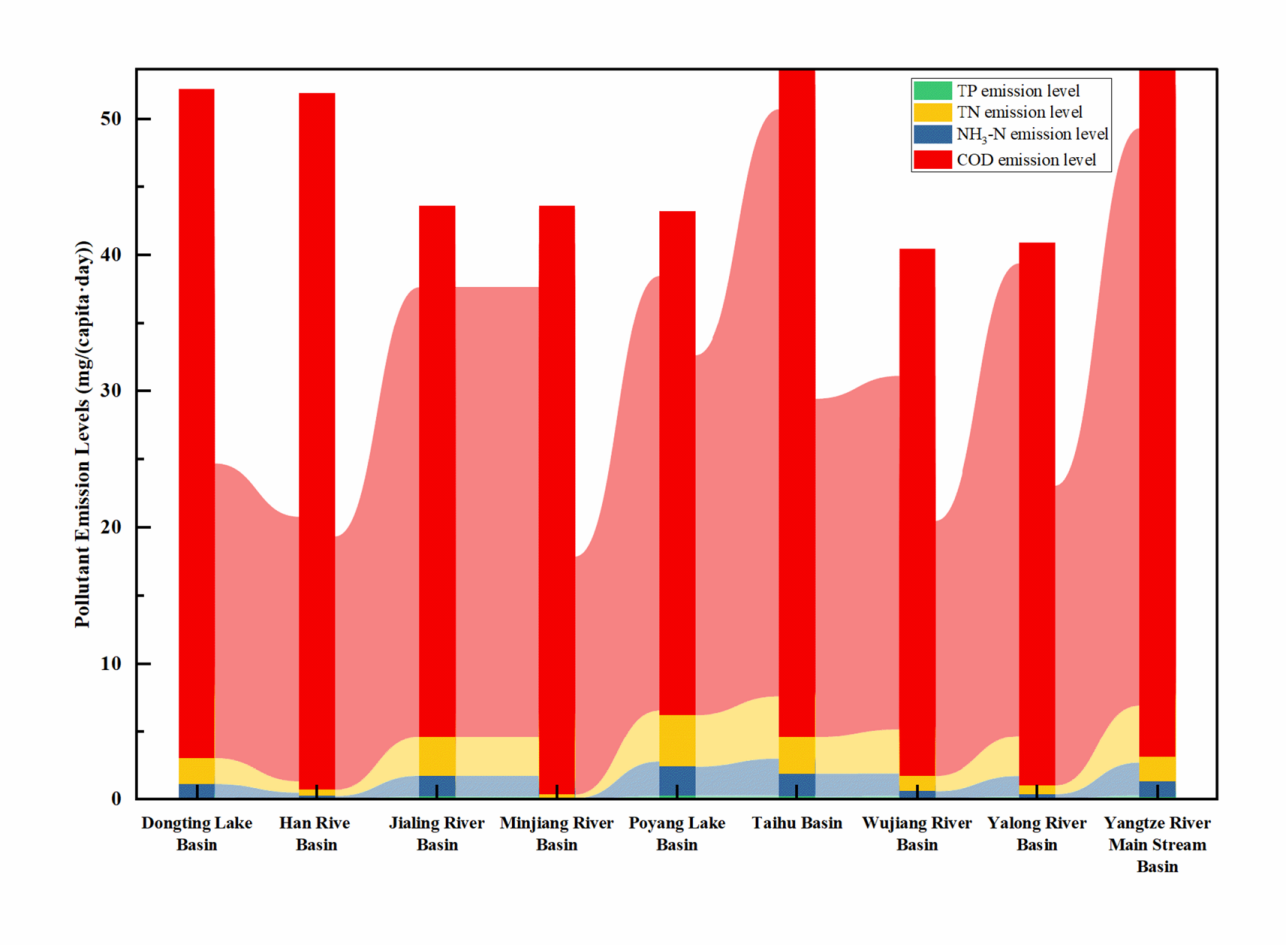
Emissions of pollutants in rural domestic sewage in the watersheds of the first-order tributaries of the Yangtze River.

The levels of pollutant emissions from rural sewage in the Yangtze River Basin are low. Compared with the emissions of urban domestic sewage in China, the emission levels account for only 3/50 to 1/10. As shown in Fig. 3, the average emission levels of the chemical oxygen demand (COD), NH_3_-H, total nitrogen (TN), and total phosphorous (TP) from rural domestic sewage in the Yangtze River Basin are 27.50 mg/(capita·day), 1.53 mg/(capita·day), 2.74 mg/(capita·day), and 0.22 mg/(capita·day), respectively, with mean standard errors of 0.64, 0.07, 0.13, and 0.01, respectively. These findings indicate that the emission levels of pollutants from rural domestic sewage in the Yangtze River Basin are primarily concentrated at lower levels. The modal values of the COD, NH_3_-H, TN, and TP emission levels, which are 29.73 mg/(capita·day), 2.51 mg/(capita·day), 4.58 mg/(capita·day), and 0.34 mg/(capita·day), respectively, further confirm the low emission levels of pollutants from rural domestic sewage as well as the variability in the NH_3_-H, TN, and TP emission levels across different cities. Range and dispersion analysis of the overall pollutant emission levels revealed that the ranges for COD, NH_3_-H, TN, and TP emissions were 28.49 mg/(capita·day), 3.67 mg/(capita·day), 5.22 mg/(capita·day), and 0.52 mg/(capita·day), respectively, with standard deviations of 7.12 mg/(capita·day), 0.82 mg/(capita·day), 1.39 mg/(capita·day), and 0.10 mg/(capita·day), respectively. This further reflects that the COD emission levels from rural domestic sewage in the Yangtze River Basin are relatively concentrated, while the NH_3_-H and TN emission levels are relatively dispersed, indicating that the pollutant emission levels are concentrated mainly around the mean, but the variability in NH_3_-H, TN, and TP is greater.

#### Characteristics of regional water quantity emissions in the Yangtze river basin

**Fig. 4:**
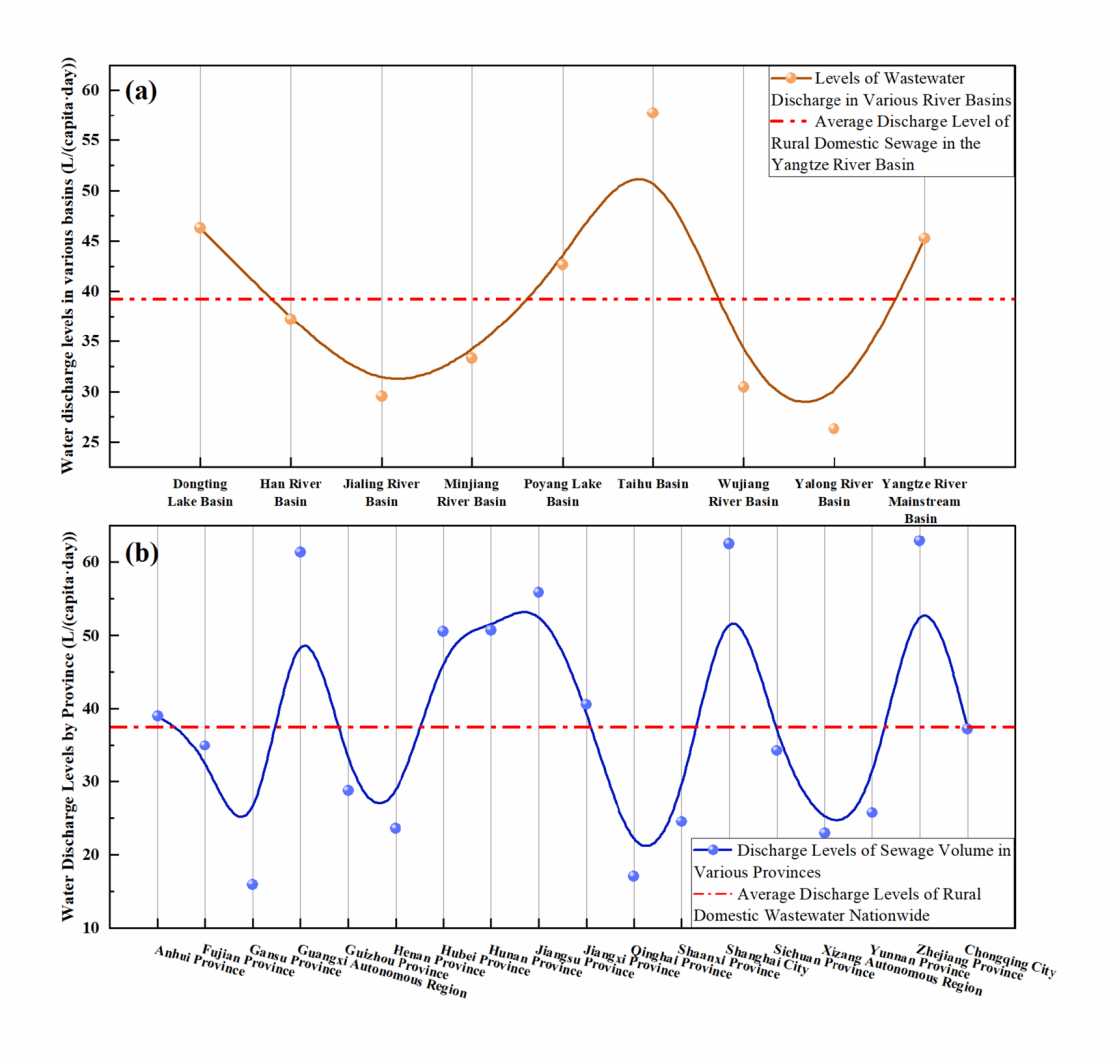
Rural wastewater discharge volumes in the primary watersheds (a) and various provinces (b) of the Yangtze River Basin.

Regional differences often indicate variations in ethnic composition, economy, and management, leading to differences in rural domestic sewage discharge levels. Significant disparities in rural domestic sewage discharge exist across different administrative regions within the Yangtze River Basin, as illustrated in Fig. 4. The upstream boundary provinces, particularly those in the western and northern regions, exhibit the lowest levels of rural domestic sewage discharge in the basin, with Gansu Province having a mean discharge level of only 15.95 L/(capita·day), followed by Qinghai Province, the Tibet Autonomous Region, Henan Province, and Shaanxi Province^36^. The rural domestic sewage discharge levels in the midstream provinces are at intermediate levels, exceeding those in Gansu Province by more than 100%, with average discharge levels of 33.97 L/(capita·day) and 37.26 L/(capita·day) for Sichuan Province and Chongqing Municipality, respectively. The downstream regions have the highest levels of rural domestic sewage discharge, with the average sewage discharge level in Zhejiang Province reaching as high as 60.36 L/(capita·day), nearly doubling that of Sichuan Province and almost tripling that of Gansu Province^37^.

Overall, the rural domestic sewage discharge levels in the Yangtze River Basin gradually increase from upstream to downstream across the secondary river basin regions. The rural sewage discharge levels in the basins of the upstream tributaries are the lowest, with the average sewage discharge level in the Yalong River Basin reaching 26.32 L/(capita·day). The average rural domestic sewage discharge level in the midstream Han River Basin is 37.21 L/(capita·day), while the downstream Taihu Lake Basin exhibits the highest average discharge level at 57.72 L/(capita·day), exceeding that of the Yalong River Basin by 1.19 times. Discrete analysis, as shown in Table 1, reveals that the rural sewage discharge levels in the basins of the upstream tributaries, such as the Yalong, Min, and Wu Rivers, are concentrated around the average value, whereas the discharge levels in the midstream tributary basins, including the Han River and the main stream of the Yangtze River, are relatively dispersed around the average value.

**Table 1:** Table of Concentration, Dispersion, and distribution of rural domestic sewage discharge levels in the Yangtze river Basin.

Type		WW Dis. Lvl.	COD Dis. Lvl.	NH₃-*N* Dis. Lvl.	TN Dis. Lvl.	TP Dis. Lvl.
Central tendency	Average value	39.24	27.5	1.53	2.74	0.22
	Median	39.5	27.08	1.5	2.72	0.22
	Mode	48.77	29.73	2.51	4.58	0.34
	Sum	4826.91	3383.09	187.7	337.41	27.48
Discrete	Standard deviation	13.8	7.12	0.82	1.39	0.1
	Variance	190.33	50.66	0.68	1.93	0.01
	Scope	63.78	28.49	3.67	5.22	0.52
	Minimum value	15.01	17.27	0.06	0.27	0.04
	Maximum value	78.79	45.76	3.73	5.49	0.56
	Average standard error	1.24	0.64	0.07	0.13	0.01
Distribution	Skewness	0.34	0.72	0.09	0.01	0.31
	Deviation standard error	0.22	0.22	0.22	0.22	0.22
	Kurtosis	−0.33	0.09	−0.8	−1.01	0.22
	Kurtosis standard error	0.43	0.43	0.43	0.43	0.43

*WW Dis. Lvl.*Wastewater discharge level, *COD Dis. Lvl.* Chemical oxygen demand discharge level, *NH₃-N Dis. Lvl.* Ammonia nitrogen discharge level, *TN Dis. Lvl.* Total nitrogen discharge level, *TP Dis. Lvl.* Total phosphorus discharge level

#### Regional water quality emission characteristics in the Yangtze river basin

**Fig. 5:**
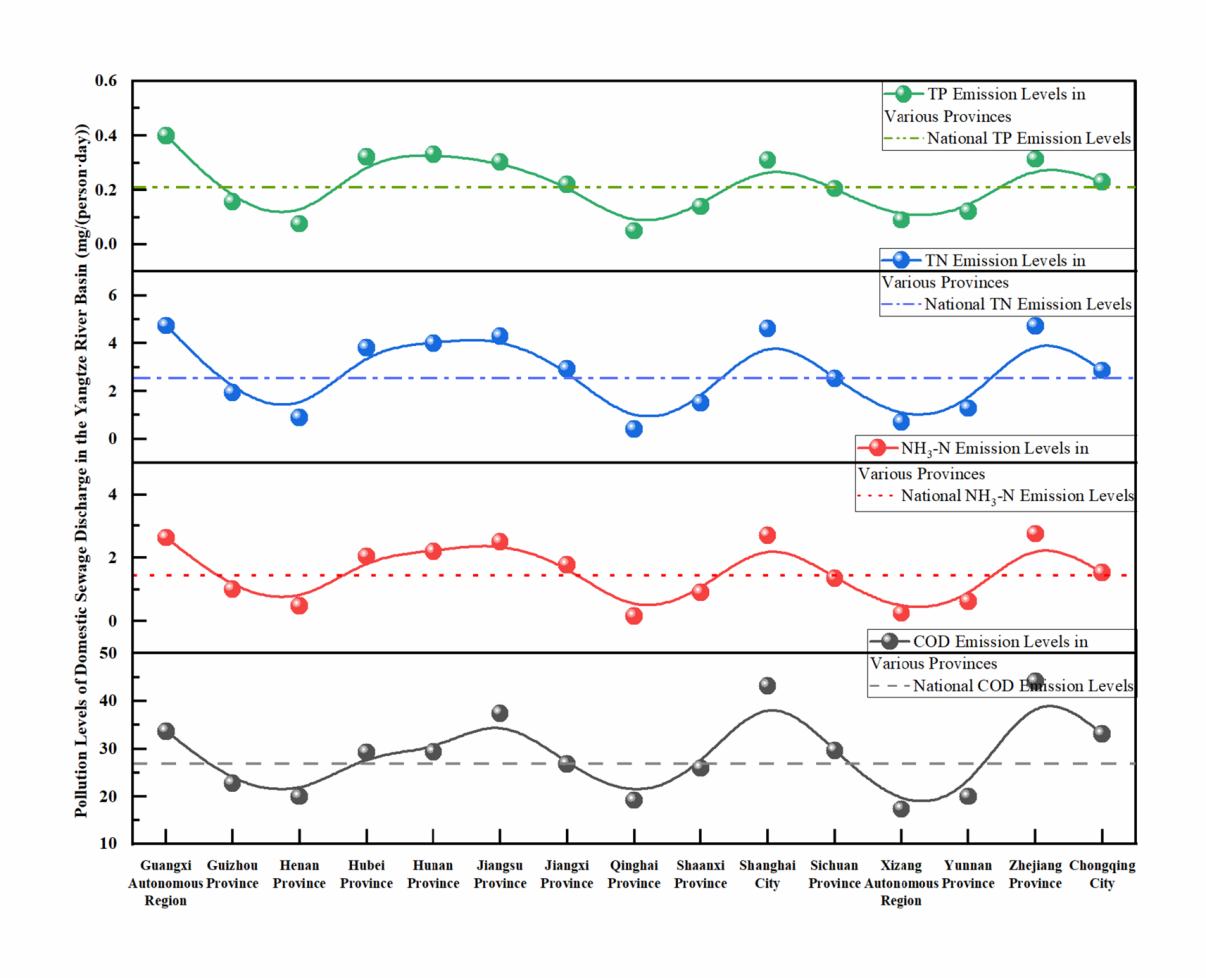
Levels of pollutant emissions in rural domestic sewage in various provinces of the Yangtze River Basin.

There are significant differences in the levels of pollutant emissions from rural domestic sewage among the different administrative regions in the Yangtze River Basin, which are not correlated with the sewage discharge volume. As shown in Fig. 5, the levels of pollutant emissions in rural sewage in the boundary provinces of the Yangtze River Basin, such as the Tibet Autonomous Region, Gansu Province, Qinghai Province, and Henan Province, are relatively lower than those in other provinces within the basin. For instance, the emission level of the COD in the Tibet Autonomous Region is only 17.27 mg/(capita·day), while the pollutant levels of ammonia nitrogen (NH_3_-N), TN, and TP in Qinghai Province are 0.16 mg/(capita·day), 0.41 mg/(capita·day), and 0.05 mg/(capita·day), respectively, all of which are at the minimum emission levels^38^. The pollutant emission levels in the midstream provinces, such as Jiangxi Province, Hubei Province, Hunan Province, and Sichuan Province, are close to the average emission levels for the entire Yangtze River Basin, with COD emission levels ranging from 26.76 to 29.36 mg/(capita·day), NH_3_-N levels ranging from 1.52 to 2.19 mg/(capita·day), TN levels ranging from 2.85 to 4.00 mg/(capita·day), and TP levels ranging from 0.20 to 0.33 mg/(capita·day)^39,40^. In contrast, the downstream provinces, such as Jiangsu Province and Zhejiang Province, show significant discrepancies in pollutant emission levels, with the COD, NH_3_-N, and TN emission levels exceeding those of the upstream boundary provinces by 1.37, 15.8, and 9.92 times, respectively, and surpassing the levels of the midstream provinces by 1.4, 1.02, and 0.81 times, respectively^41^. However, the TP emission levels are notably lower than those of the midstream provinces, being 0.94 times the midstream TP emission levels and exceeding those of the upstream source provinces by 5.08 times. These observations may be attributed to the higher population density in the midstream regions, along with poorer economic conditions than in the downstream regions, resulting in lower per capita water consumption and poor dissemination of water-saving devices, which amplifies the concentration effect of phosphorus-containing pollutants^42^. The per capita sewage discharge levels in the midstream region indirectly support this hypothesis.

**Fig. 6:**
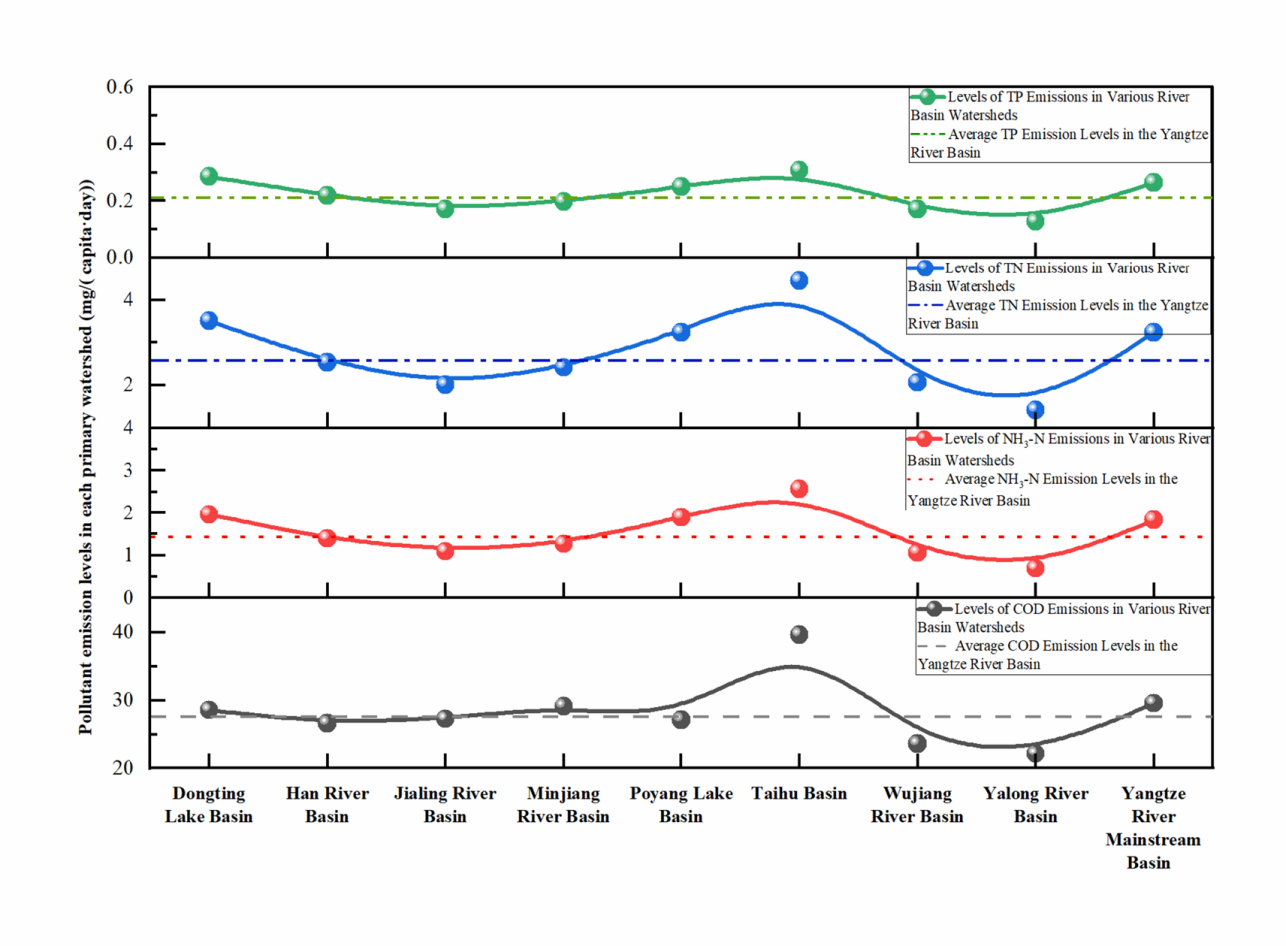
Pollutant discharge levels for rural domestic wastewater in the tributary basins of the Yangtze River Basin.

As shown in Fig. 6, the levels of pollutant emissions in rural domestic sewage in the upper tributary basins of the Yangtze River are relatively low compared to the middle to lower reaches. The COD and NH_3_-N emission levels in the Yalong River Basin and Wu River Basin were the lowest at 22.18 mg/(capita·day) and 0.69 mg/(capita·day) and 23.65 mg/(capita·day) and 1.06 mg/(capita·day), respectively. Conversely, the pollutant emission levels in the downstream Taihu Lake Basin are the highest, with the COD, NH_3_-N, TN, and TP emission levels reaching 39.60 mg/(capita·day), 2.57 mg/(capita·day), 4.45 mg/(capita·day), and 0.31 mg/(capita·day), respectively. In the midstream regions, such as the Han River Basin and Poyang Lake Basin, the pollutant emission levels fluctuate significantly; for example, the COD emission levels in the Han River Basin and Poyang Lake Basin were lower than those in the upstream Jialing River Basin and Min River Basin, but the NH_3_-N, TN, and TP emission levels were higher than those in the upstream Jialing River and Min River Basins. The pollutant levels in the downstream basins exceeded those in the upstream basins by 0.78, 2.71, 2.14, and 1.4 times for COD, NH_3_-N, TN, and TP, respectively.

### Standardized pollution load analysis

The discharge of rural domestic sewage in China is characterized by randomness and instability. Rural domestic sewage flows into rivers along with surface runoff, which can contaminate river water at any time. Equivalent pollution load analysis can reflect the impact of rural domestic sewage on maintaining the water quality goals of rivers. The average runoff coefficient in the Yangtze River Basin over time is 0.51 (0.4 for the Taihu Basin)^43–45^. The results of the analysis indicate that the discharge of rural domestic sewage pollutants in the Yangtze River Basin has a relatively weak effect on maintaining the Class II water quality functional objectives of the first-level tributaries or the main stream of the Yangtze River within the watershed. The equivalent pollution loads for maintaining these objectives due to rural domestic sewage pollutants across various provinces range from 0.00 to 10.80 × 10^−2^. Specifically, the equivalent load from COD emissions varies between 0.00 and 4.44 × 10^−2^, that from NH_3_-N emissions is between 0.00 and 6.11 × 10^−2^, that for TN emissions is between 0.00 and 10.80 × 10^−2^, and that for TP emissions is between 0.00 and 4.51 × 10^−2^. The median ratios of the equivalent pollution loads from rural domestic sewage pollutants within the jurisdiction of the Yangtze River Basin that are needed to maintain the water quality functional objectives for the first-level tributaries or the main stream are as follows: 0.13% COD, 0.22% NH_3_-N, 0.41% TN, and 0.17% TP. These findings indicate that the influence of rural domestic sewage discharge on the water quality of these water bodies can be considered negligible.

**Fig. 7:**
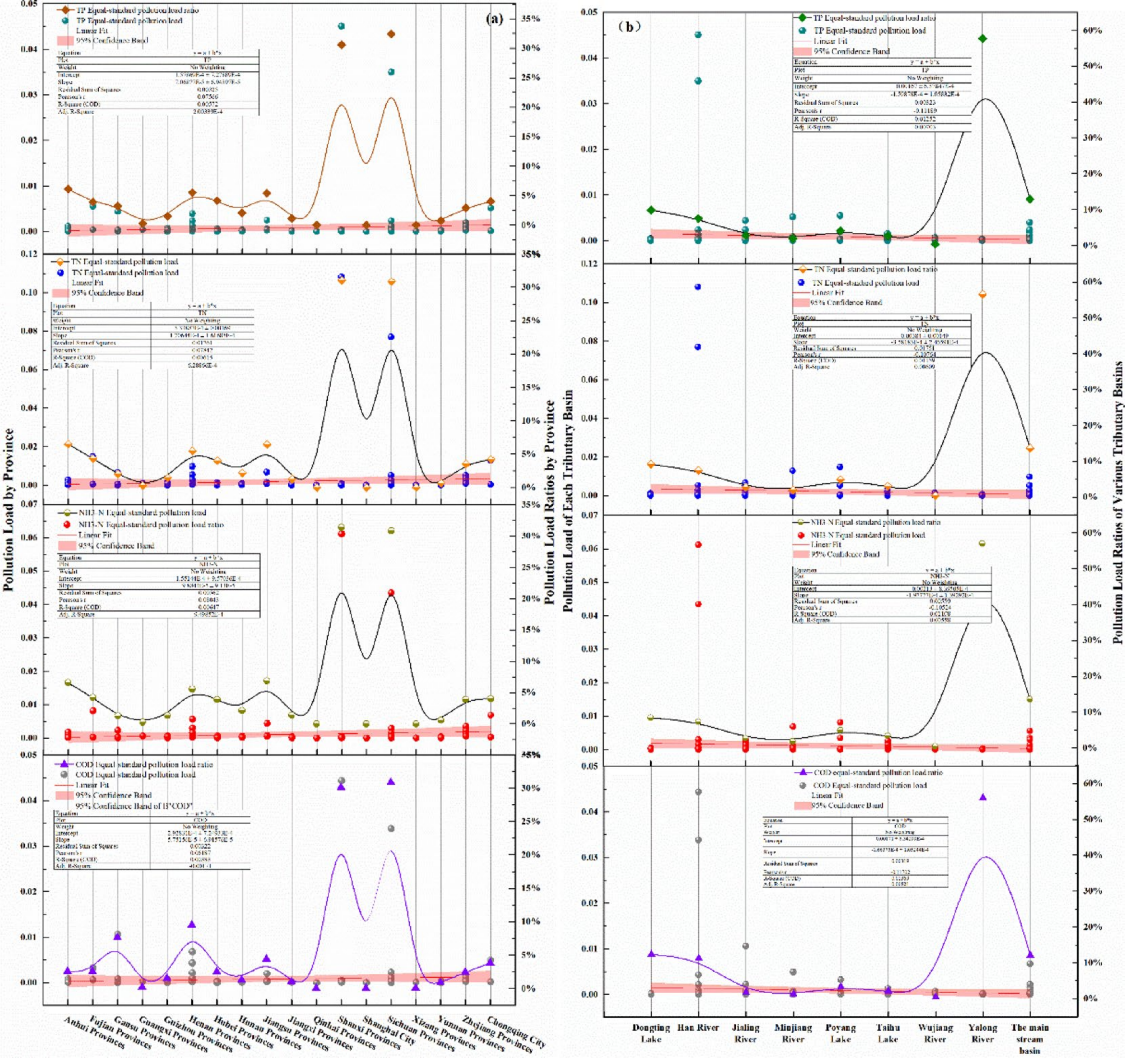
Load situation for each province in the Yangtze River Basin (a) and for each watershed (b).

Specifically, the impact of pollutants found in rural domestic sewage varies across different administrative regions on the first-level tributaries or the main stream of the Yangtze River, as illustrated in Fig. 7(a). In provinces such as Sichuan, Shaanxi, Henan, and Gansu, the maximum equivalent pollution load ratios due to rural domestic sewage discharge affecting the water quality functional objectives range from 30.84% to 32.41%, 30.03% to 31.37%, 5.48% to 9.49%, and 1.30% to 7.61%, respectively. The differences in pollution load pressures from rural domestic sewage pollutants between Sichuan and Shaanxi are minimal, whereas the disparities in Henan and Gansu are notable, with COD contributing the greatest equivalent pollution load ratio to maintaining the water quality objectives in the first-level tributaries.

As shown in Fig. 7(b), the Yalong River Basin, the main stream of the Yangtze River Basin, the Dongting Lake Basin, and the Han River Basin are the regions under the highest pressure from rural domestic sewage pollutant discharge on maintaining water quality functional objectives. The equivalent pollution load ratios for the Han River Basin ranged from 57.22% to 58.39%, those for the main stream of the Yangtze River ranged from 13.14% to 14.23%, those for the Jialing River Basin ranged from 5.43% to 11.76%, and those for the Min River Basin ranged from 5.39% to 6%.

### Analysis of the factors influencing rural domestic sewage discharge levels

**Fig. 8:**
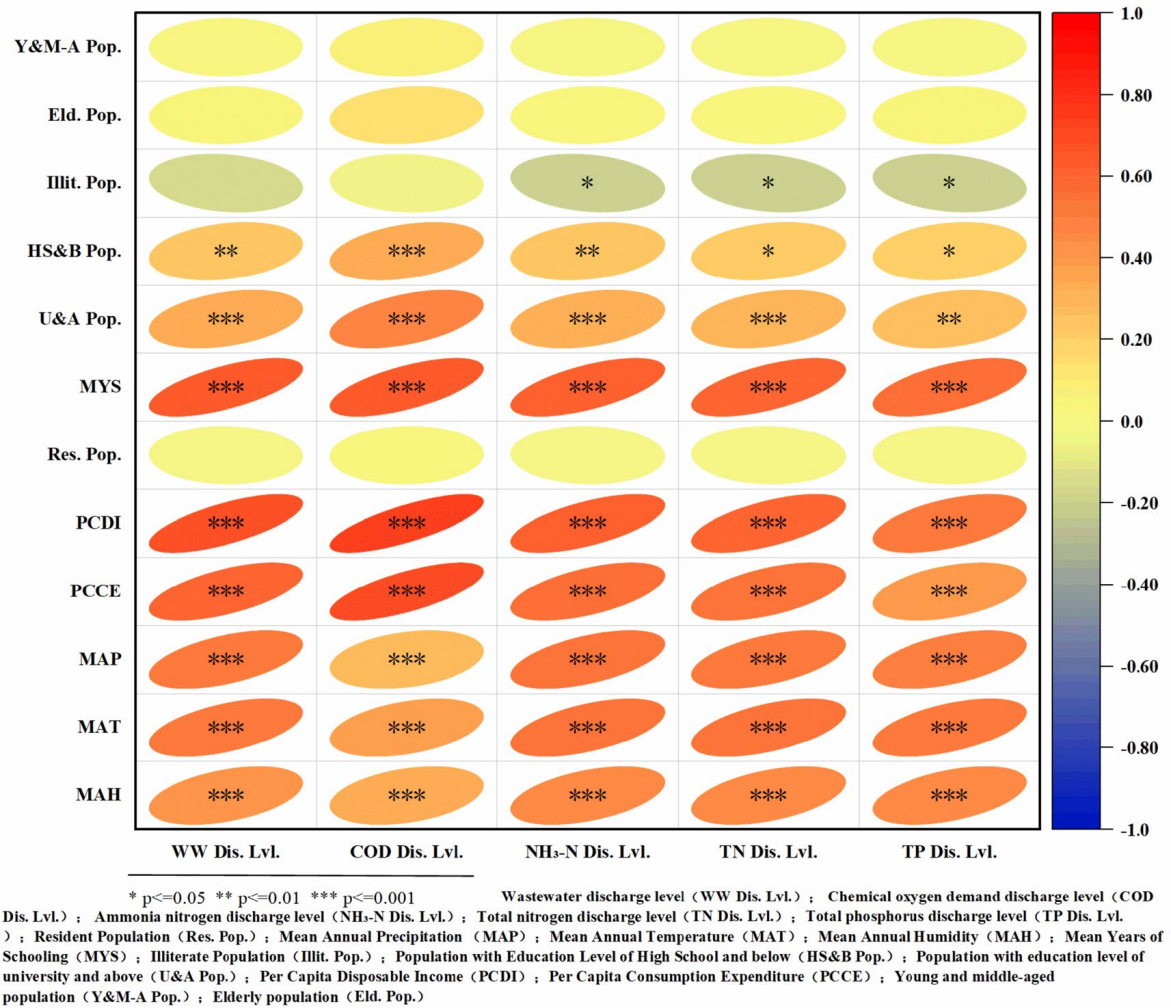
Correlation analysis heatmap of factors influencing rural domestic sewage discharge levels in the Yangtze River Basin.

According to the results of the Pearson correlation analysis results, factors such as educational level, disposable income, annual average rainfall, annual average temperature, and annual average humidity are significantly correlated with the rural domestic sewage discharge levels in the Yangtze River Basin. As shown in Fig. 8; Table 2, there is a significant negative correlation between annual average rainfall and rural domestic sewage discharge levels, with correlation coefficients (r) of −0.472 for water quantity discharge levels and − 0.512 to −0.419 for water quality discharge levels, with a significance level of *p* < 0.001. Rainfall has a negative interaction with temperature (correlation coefficient r of −0.468) and humidity (correlation coefficient r of −0.493); as rainfall increases, temperature and humidity tend to decrease, leading to greater suppression of water demand by villagers, which consequently reduces the discharge of domestic sewage. Additionally, rainfall occurrence suppresses the daily behaviours of villagers, which in turn inhibits habits that are related to water use for dietary and personal hygiene purposes, thereby lowering the discharge levels of rural domestic sewage. Both the annual average humidity and the annual average temperature are significantly positively correlated with the discharge levels of rural domestic sewage^4,46^. The correlation coefficient for the annual average temperature with water quantity discharge levels is *r* = 0.535, and that with water quality discharge levels is *r* = 0.390 to 0.521, with a significance level of *p* < 0.001; the correlation coefficient for the annual average humidity with water quantity discharge levels is *r* = 0.441, and that with water quality discharge levels is *r* = 0.356 to 0.470, with a significance level of *p* < 0.001. Temperature and humidity significantly affect the dietary structure and water usage behaviours of rural residents, notably altering the intake of high-fat and high-protein foods under high-temperature and high- humidity conditions, whereas humid and hot environments also increase the frequency of cleaning, affecting sewage discharge levels^47–49^.

Factors such as years of education, educational level, disposable income, and consumption expenditures are significantly positively correlated with the discharge levels of rural domestic sewage. The number of years of education exhibits a correlation coefficient (r) of 0.633 with water quantity discharge levels and 0.558 to 0.637 with water quality discharge levels, with a significance level of *p* < 0.001. The correlation coefficient (r) of individuals with high school education or below is 0.330 for water quantity discharge levels and 0.257 to 0.296 for water quality discharge levels, with a significance level of *p* < 0.05. The number of illiterate individuals is negatively correlated with the discharge levels of rural domestic sewage, but it is not significant, with a correlation coefficient (r) of −0.174 for water quantity discharge levels and − 0.072 to −0.229 for water quality discharge levels, with a significance level (p) ranging from 0.012 to 0.432. Factors related to individuals with a college education or higher are positively correlated with the discharge levels of rural domestic sewage, but the correlation coefficient (r) is not significant: 0.143 for water quantity discharge levels and 0.064 to −0.140 for water quality discharge levels, with a significance level of *p* > 0.05. This finding indicates that education significantly affects the awareness, economy, and technological content of rural residents’ lives; higher educational levels correlate with a greater emphasis on hygiene and health, leading to more pronounced changes in dietary structure, which in turn helps to alter domestic sewage discharge levels^50–54^. However, after a college education level or higher is reached, although a positive correlation exists, it is no longer significant. The overall average income of rural residents with a college degree or higher in the Yangtze River Basin is at a standstill, rendering breakthroughs difficult and indicates that the economy is a decisive factor driving the increase in sewage discharge^23^. The correlation between a high school education or below and per capita disposable income is (correlation coefficient *r* = 0.610, significance level *p* < 0.001), and per capita consumption expenditure (correlation coefficient *r* = 0.337, significance level *p* < 0.05) supports this hypothesis.

**Table 2:** Correlation of influencing factors on rural domestic sewage discharge levels in the Yangtze river Basin.

Factors	Pearson correlation					*P* value				
	WW Dis. Lvl.	COD Dis. Lvl.	NH₃-*N* Dis. Lvl.	TN Dis. Lvl.	TP Dis. Lvl.	WW Dis. Lvl.	COD Dis. Lvl.	NH₃-*N* Dis. Lvl.	TN Dis. Lvl.	TP Dis. Lvl.
PRs under 60	0.021125	0.0967405	0.00831	0.0037247	0.0178144	0.816599	0.2871255	0.9273154	0.9673859	0.8449468
60 + Res. Pop.	0.064335	0.1482915	0.05873	0.0539395	0.0780214	0.4795933	0.1016506	0.5187626	0.5534879	0.3910178
Illit. Pop.	−0.172305	−0.0790669	−0.2000075	−0.2009821	−0.2078399	0.05985628	0.39066441	0.02850827	0.02772787	0.02273401
HS&B Pop.	0.25531319	0.3580592	0.24022226	0.23380339	0.20356298	0.00488773	< 0.0001	0.00822296	0.01016676	0.02574852
U&A Pop.	0.35556641	0.48649542	0.323929	0.31061023	0.27263658	< 0.0001	< 0.0001	0.00030721	0.00055488	0.00259011
MYS	0.6523167	0.6467973	0.6227837	0.6079514	0.5795111	< 0.0001	< 0.0001	< 0.0001	< 0.0001	< 0.0001
Res. Pop.	−0.03249	0.0444716	−0.046119	−0.049893	−0.03854	0.7257591	0.6310531	0.6184472	0.5899875	0.6773107
PCDI	0.6912297	0.7569179	0.6293082	0.6119205	0.5255197	< 0.0001	< 0.0001	< 0.0001	< 0.0001	< 0.0001
PCCE	0.6072951	0.7008741	0.567532	0.5511909	0.4194949	< 0.0001	< 0.0001	< 0.0001	< 0.0001	< 0.0001
MAP	0.5299672	0.2993845	0.5459335	0.521431	0.5057795	< 0.0001	0.0007684	< 0.0001	< 0.0001	< 0.0001
MAT	0.5309012	0.3976707	0.54736	0.5439121	0.5347019	< 0.0001	< 0.0001	< 0.0001	< 0.0001	< 0.0001
MAH	0.432539	0.352694	0.4669469	0.4682499	0.4690297	< 0.0001	< 0.0001	< 0.0001	< 0.0001	< 0.0001

*PRs under 60* permanent residents under the age of 60,*60 + Res. Pop.* Resident Population Aged 60 and Above, *Illit. Pop.* Illiterate Population, *HS&B Pop.* Population with Education Level of High School and below, *U&A Pop.* Population with education level of university and above, *MYS* Mean Years of Schooling, *Res. Pop.* Resident Population, *PCDI*Per Capita Disposable Income, *PCCE*Per Capita Consumption Expenditure, *MAP*Mean Annual Precipitation, *MAT*Mean Annual Temperature, *MAH* Mean Annual Humidity

## Conclusion

The main findings and recommendations are as follows:

1. The current levels of rural domestic wastewater discharge in the Yangtze River Basin are low, which shows a significant gap compared with the urban domestic wastewater discharge levels in China, developed countries in Europe, and select developing nations. It is recommended that the government strengthen the analysis of the main driving factors influencing rural domestic wastewater discharge levels during the implementation of rural wastewater treatment efforts, which can help ensure the long-term effectiveness of these initiatives.
2. There are notable differences in the rural domestic wastewater discharge levels across the Yangtze River Basin, particularly in economically underdeveloped upstream regions where the discharge levels may be in their initial phase. The water quality pollution pressure associated with maintaining the water function targets of the first-level tributaries of the Yangtze River is particularly notable. With the implementation of poverty alleviation actions in rural China leading to significant changes in social factors, it is essential to continuously monitor how these changes impact the ability to maintain water quality targets in rivers.
3. This research focuses on the characteristics and influences of rural domestic wastewater at the macroscale, such as in large river basins and regions, but studies on the discharge characteristics and impacts of specific areas and rivers are lacking. This is especially true in small-scale regions where topographical and climatic differences are considerable. Therefore, this study may not comprehensively, in depth, or specifically reflect the concrete impacts of rural domestic wastewater discharge levels on river water quality under complex interactions.

## Data Availability

All original data sources in this study are also included in the “Data Sources and Processing” section of previously published articles.- The datasets generated during and/or analysed during the current study are available from the corresponding author on reasonable request.
